# 加强血液重症单元建设全面改善血液危重症患者预后

**DOI:** 10.3760/cma.j.cn121090-20241225-00592

**Published:** 2025-01

**Authors:** 豫 胡, 耀辉 吴

**Affiliations:** 华中科技大学同济医学院附属协和医院血液病学研究所，武汉 430022 Institute of Hematology, Union Hospital, Tongji Medical College, Huazhong University of Science and Technology, Wuhan 430022

**Keywords:** 血液专科重症单元, 危重症, 恶性血液肿瘤, Hematology intensive care unit, Critical illness, Hematological malignancy

## Abstract

近年来，恶性血液病患者的生存期明显延长，血液恶性肿瘤相关严重并发症的发生率亦相应升高，且往往病情变化急骤，与原发病交错重叠。设立血液专科重症单元（hematology intensive care unit, HCU）对于早期识别、集中监护式管理血液危重症患者，发挥专科优势、精准把控病情，提高血液科诊治水平具有重要临床意义。我们建议具备相应条件的血液中心积极建立HCU，全面改善血液危重症患者的预后。

## Abstract (en)

The survival time of patients with hematological malignancies has improved significantly in recent years; however, the incidence of severe complications has also increased and may escalate rapidly alongside the progression of the primary disease. The establishment of a hematology intensive care unit（HCU）is of great clinical significance for early identification, centralized monitoring and management of hematological critically ill patients, leveraging the advantages of specialties, accurately controlling the condition, and improving the level of diagnosis and treatment in hematology. It is recommended that blood centers with adequate resources actively establish HCUs to enhance the prognosis of patients with hematological critical illnesses.

随着医学技术的飞速发展，血液病的诊断与治疗已取得长足进步，但血液危重症患者的救治仍面临严峻挑战。血液危重症患者病情复杂、进展迅猛，亟需在大量医疗资源支持下快速判断、及时处理，为其度过危险期，进行下一步治疗创造机会[1]。据统计，血液恶性肿瘤（hematological malignancy，HM）患者在确诊1年内转入重症监护病房（intensive care unit，ICU）的比例达到13.9%，其中急性白血病患者转入ICU的比例高达22.5%[2]。与此同时，HM患者中老年患者比例逐年增加，且随着新型靶向及免疫治疗的积极应用和推广，相关合并症更复杂，危重症发病率明显上升。尽管ICU在重症患者救治中扮演着关键角色[3]，但血液危重症患者的诊断和治疗都具有较强的专科特征，ICU医师恐难给予及时、准确的专科处置[4]–[5]；另有部分血液科患者疾病转为危重症风险高但生命体征尚稳定，分散在各个普通病房，不利于加强监护、病情变化时及时处理。在此背景下，血液重症病房（hematology intensive care unit，HCU）的创立显得尤为必要。HCU是由掌握血液专科知识并接受急救、重症医学等诊疗技术培训的医护团队，基于多学科团队（multi-disciplinary team，MDT）协作的诊疗理念，利用先进的监护设备、诊疗手段和高级生命支持措施对血液病危重症患者实施专科化、目标化精准诊疗的医疗单元。

目前国内外恶性血液病患者五年生存率有明显差异（国内约40%，国外超过60%），血液危重症患者救治成功率不同是其原因之一。2015年新英格兰医学杂志曾统计血液科危重症转入综合ICU患者的死亡率达96%，其中部分原因是血液危重症患者按照生命体征不稳定指标转入时病情相对过重，救治难度太高[6]。另有一部分血液科患者病情风险极高但生命体征尚稳定，未达到转入ICU的标准，一旦出现病情变化时进展迅猛、变化急骤，会在极短的时间内转变为危重症且预后极差。此类患者在HCU集中管理、密切监护，病情变化时能够及时给予专科化针对性处置和强有力的生命体征支持，以阻止其向危重症患者转化，减少血液危重症患者的比例，也为后期治疗创造更好的条件。既往无HCU时，此类患者分散在普通病房，当出现生命体征不稳定时不能及时发现处置；转入ICU时又因医师血液专科知识的匮乏使处理的针对性有所欠缺，此类血液科危重症患者的原发病和并发症往往互相关联、交错重叠，进一步增加了救治的难度。为探索新的血液危重症治疗模式，华中科技大学附属协和医院血液病学研究所2011年底率先在全国范围内设立HCU。本中心HCU成立12年以来，共收治3 836例血液科危重患者，与此同时，血液科每年转入综合ICU的危重症患者比例下降90%，仅10%极危重患者需进行转诊；采取本模式后，本中心血液危重症患者死亡率可下降至50%左右，较以往有明显改善。因此，在血液科危重症救治层面上，HCU的设立具有重要意义：有利于优化诊疗流程、整合专科资源，精准把控血液病患者病情变化；抓住救治黄金时机，进而切实提高救治成功率，降低死亡率；破除传统ICU救治血液危重症的局限性。

经过多年的探索与实践，我们充分体会到血液危重症患者的专科特性及管理难度。目前国内外仅有部分关于血液危重症患者预后因素的研究[7]–[8]，但尚缺乏全面系统的专家共识指导此类患者的综合诊疗与管理。我们组织国内多个血液重症中心的专家组成员，参考国内外ICU及血液科的建设指南，共同制定了《血液专科重症单元危重症患者诊疗和管理中国专家共识（2025年版）》，旨在为血液科危重症患者的管理提供切实可行的建议，以提高血液危重症的救治水平。

一、HCU的救治优势

HCU的功能与定位与ICU存在显著差异，其并非ICU的分支。HCU建立在血液科病房基础之上，主要面向血液科危重患者，其治疗与监护措施具有鲜明的专科特色。在现代医疗体系中，HCU的成立标志着血液危重症患者的救治工作迈入了一个全新的阶段。HCU具备以下四个优势：

1. 发挥专科优势，精准把控病情：血液危重症患者具有免疫缺陷、粒细胞缺乏、贫血、血小板低、凝血功能障碍等专科特点，易出现重症感染、弥散性血管内凝血、血栓性血小板减少性紫癜、肺栓塞、高白细胞血症、急性溶血等严重并发症[9]–[11]，需要及时进行专科处理。此外，对于初治急性白血病及噬血细胞综合征等血液病患者而言，病因治疗是阻止其进展为危重症的关键，也需尽快开始血液专科治疗[12]。HCU的专科团队凭借丰富的临床经验和专业知识，能够更准确地掌握上述血液病患者的专科病情，在兼顾危重症的救治时制定出更具针对性的综合治疗策略，尽快启动血液专科治疗，从而有效阻遏病情的进一步恶化。

2. 依托MDT协作，专业高效处理并发症：HCU践行MDT协作救治模式，血液专科、ICU专科医师携手，搭配急诊科、感染科、肾内科、呼吸科等学科力量。各学科发挥专业所长、紧密配合，在诊断、治疗与护理各环节协同互助，病情变化迅速响应、精准施治，从而降低血液危重症患者死亡率。

3. 灵活分流患者，提升救治效率：HCU显著提高了血液危重症患者转入ICU接受专业治疗的比例，有效缓解了重症病房床位需满足全院重症需求而有时相对紧张的问题，让部分生命体征稳定的血液科危重症患者更早期接受严密监护，缩短了从发现病情转变到及时给予对应措施的时间，极大地提升了整体救治效率。HCU的救治经历能够准确筛选出需要进一步转入综合ICU进行更高级别生命支持的极危重症患者；同时，HCU也可充当血液病患者从综合ICU平稳过渡到血液科普通病房的桥梁，确保患者在不同治疗阶段无缝衔接。这种良好的互动机制，不仅优化了医疗资源的配置，还提升了整个医疗体系的协同作战能力。

4. HCU实践成果显著：目前，武汉协和医院血液病学研究所HCU成立十余年的数据显示，HCU成立后血液科危重症患者的收治数量、比例及治疗成功率均呈现逐年上升趋势。对于危重症初治急性髓系白血病患者而言，在转入HCU后接受第1个疗程维奈克拉联合去甲基化药物治疗，高达75%的患者可达到完全缓解（CR）或CR伴不完全血液学恢复（CRi），平均住院时间为27.3 d[13]；且此类患者的死亡率及综合ICU转诊率均呈现明显下降趋势。

二、HCU模式的建设与创新

HCU作为集血液专科与急救、重症医学于一体的专业医疗单元，其建设与推广在现代医疗体系中占有举足轻重的地位。

1. 打造多学科协作团队，提升救治科研实力：HCU的建设是医院在血液病领域专业实力的重要体现。通过血液科、急诊科、重症医学科、感染科等多个学科的紧密配合，HCU形成了一个高效协作的医疗团队，这一模式打破了传统科室间的壁垒，使患者在面临复杂病情时能够迅速获得来自多个领域专家的会诊和精准治疗。上述多学科协作的模式不仅提高了危重症患者的救治成功率，还为交叉科研提供了更多的合作机会和研究方向，可推动血液病和危重症医学领域的科研进步。同时，HCU的建设也提升了医院的整体医疗水平，增强了医院在业界和患者中的声誉和影响力，可为患者提供更加可靠、专业的医疗服务。

2. 配备先进设备设施，提供强有力的生命支撑：HCU的建立往往伴随着先进医疗设备的引进和现代化医疗设施的建设。这些医疗设备包括但不限于多功能监护仪、无创呼吸机、除颤仪、细胞单采机、血浆置换机、血液透析机等，能够实时提供监护数据，为医师及护士提供了更加准确、便捷的诊疗手段，并针对不同危重症情形提供替代治疗或生命支持治疗。高标准的环境设施体现于中央监护站、科学设计的床单位等，为患者提供了更安全、舒适的治疗环境，提升了患者的就医体验和康复效果。

3. 规范诊疗流程，保障权益与效益：为确保医疗服务的高效与安全，HCU强调临床路径管理和标准化诊疗流程的应用。通过制定严格的临床路径和规章制度，HCU可确保每例患者得到科学合理的治疗。这种规范化的诊疗行为不仅有效控制了医疗成本，提高了医疗资源的利用效率，还保障了患者的合法权益，增强了医疗服务的透明度和公信力。此外，HCU还注重患者信息的保密和安全管理，确保患者的隐私权益得到充分保护。

4. 培育专业人才，加强医德医风建设：HCU还是医疗人才培养的重要基地，不仅注重医护人员的专业技能培训，还强调医德医风建设和团队协作能力的培养。通过举办学术会议、研讨会、进修班等多种形式，HCU为医护人员提供了广阔的学习和发展平台，促进了医疗人才队伍的快速成长和整体素质的提升。这些培训和学习机会不仅提高了医护人员的专业技能水平，还增强了他们的职业素养和团队协作能力，为医院的长远发展奠定了坚实的人才基础。

因此，HCU模式的建设与创新在提升危重患者救治能力、促进多学科协作、改善医疗条件、规范诊疗行为及培养专业人才等方面都具有重要意义。

三、展望

华中科技大学附属协和医院血液病学研究所2011年底率先在全国范围内设立HCU，此后哈尔滨医科大学附属第一医院、中国医学科学院血液病医院、苏州大学附属第一医院等多个医院血液科也陆续成立HCU，另有一些地市级医院也逐步创建了HCU。不久前，国家卫生健康委等八部门联合制定了《关于加强重症医学医疗服务能力建设的意见》，体现了国家对危重症患者医疗服务的重视，血液科无疑也在其中。《健康中国行动—癌症防治行动实施方案（2023—2030年）》也明确提出，到2030年我国总体癌症5年生存率要达到46.6%。在这一目标指引下，更有效地救治血液病危重症患者也成为提高血液肿瘤患者生存率的关键因素之一。在癌症防治的整体框架下，恶性血液病危重症患者的救治需要高度专业化的医疗设施和强有力的重症生命支持手段。HCU作为针对血液病危重症患者的专业救治平台，能够提供及时、专业、全面的医疗服务，对于恶性血液病危重症患者的救治至关重要。我们倡导全国所有血液医疗中心能在条件具备时建立HCU，通过及时规范的诊疗，为更多血液危重症患者带来生命的希望，为健康中国行动贡献力量！
